# Association of Non-High-Density Lipoprotein Cholesterol With Cardiometabolic Risk Factors in Patients With Hypothyroidism

**DOI:** 10.7759/cureus.64531

**Published:** 2024-07-14

**Authors:** Mohammed M Khan, Preeti Yadav, Seun Arowolo, Anne Saidu, Seyi A. Olaniyi, Parvinder Kaur, Sai Harini Chandrasekaran, Jeffrin J Varghese, Chinyere K Omeh, Roshini Vijayakumar, Mohammed Kashif, Saba Khan, Roshan Alam

**Affiliations:** 1 Basic Medical Sciences, Integral Institute of Allied Health Sciences and Research, Lucknow, IND; 2 General Medicine, Grant Government Medical College, Mumbai, IND; 3 Internal Medicine, Obafemi Awolowo University, Ile Ife, NGA; 4 Internal Medicine, V. N. Karazin Kharkiv National University, Kharkiv, UKR; 5 Medicine, Obafemi Awolowo University, Ile Ife, NGA; 6 Internal Medicine, Crimean State Medical University, Simferopol, UKR; 7 General Medicine, Government Medical College, Chennai, IND; 8 Internal Medicine, Government Medical College, Thiruvananthapuram, IND; 9 Internal Medicine, 161 Nigerian Airforce Hospital, Makurdi, NGA; 10 Biochemistry, Integral Institute of Medical Sciences and Research, Lucknow, IND

**Keywords:** non-hdl, hypothyroid, cardio-metabolic risk factors, lipid profile in hypothyroidism, dyslipidemia

## Abstract

Introduction: Non-high-density lipoprotein cholesterol (non-HDL-C) levels can increase the cardiometabolic risk factors in patients with hypothyroidism, but the findings across studies have not been consistently conclusive. The aim of this study was to find the association between non-HDL-C and cardiometabolic risk factors in patients with hypothyroidism.

Material and methods: In this case-control study, a total of 120 subjects among which 60 diagnosed hypothyroidism patients and 60 age-matched healthy controls were enrolled, aged 30-65 years. Body mass index (BMI), waist circumference (WC), and systolic and diastolic blood pressures (SBP and DBP) were measured. Thyroid-stimulating hormone (TSH), triiodothyronine (T3), thyroxine (T4), fasting blood sugar (FBS), total cholesterol (TC), triglyceride (TG), and high-density lipoprotein cholesterol (HDL-C) were estimated. Low-density lipoprotein cholesterol (LDL-C), very low-density lipoprotein cholesterol (VLDL-C), and non-HDL-C were calculated. A p-value of <0.05 was considered statistically significant.

Results: Mean of BMI, WC, FBS, TSH, TC, TG, non-HDL-C, LDL-C, VLDL-C, SBP, and DBP were significantly elevated in cases compared to controls (p<0.001). However, the mean of T3, T4, and HDL-C were significantly reduced in cases compared to controls (p<0.001). Non-HDL-C has shown a significant positive correlation with age (r=0.345, p<0.01), TC (r=0.451, p<0.01), TG (r=0.269, p<0.05), LDL-C (r=0.402, p<0.01), and VLDL-C (r=0.269, p<0.05) among cases. However, non-HDL-C has shown a significant negative correlation with HDL-C (r=-0.330, p<0.05) among cases. Non-HDL-C significantly predicted cardiometabolic risk in patients with hypothyroidism (F(13,46)=3.500, p<0.001).

Conclusion: Non-HDL-C has shown a significant association with age and lipid abnormalities in patients with hypothyroidism. Non-HDL-C significantly predicts cardiometabolic risk factors in patients with hypothyroidism.

## Introduction

Hypothyroidism is the most prevalent thyroid disorder. It can be defined as elevated thyroid-stimulating hormone (TSH) and low levels of triiodothyronine (T3) and thyroxine (T4) [1]. The prevalence of hypothyroidism varied in gender, age, and ethnic groups. The prevalence of hypothyroidism was 13.95% in mainland China, 11.7% in the United States, 3.6% in the United Kingdom, and 10.95% in India [2-5]. The prevalence of hypothyroidism is high in the general population mainly in women and elderly individuals [2,3]. 

Most hypothyroid patients have non-specific constant symptoms such as weight gain leading to obesity, fatigue, and mood swings even after treatment with levothyroxine (LT4) [6,7]. Various studies have suggested that hypothyroidism is associated with an increased risk of obesity, hypertension, type 2 diabetes mellitus (T2DM), and cardiovascular disease (CVD) [8-11]. 

Hypothyroidism affects cardiovascular function by reducing cardiac output and increasing arterial stiffness due to reduced nitric oxide and altered calcium levels. Moreover, thyroid hormones influence blood pressure through their action on the renin-angiotensin-aldosterone system (RAAS), cause normocytic normochromic anemia due to an increase in erythropoietin, and regulate genes impacting cholesterol levels, inflammation, and heart rate [12].

In this context, early screening and management of hypothyroidism is essential to reduce the cardiometabolic risk. Hypothyroidism affects the overall metabolic activities of the body and is strongly associated with hyperlipidemia and hyperglycemia [13,14]. Accumulation of epicardial adipose tissue increases the cardiometabolic risk [15]. Epicardial adipose tissue significantly increased in patients with hypothyroidism and increased the cardiometabolic risk [16]. Hypothyroidism may decrease cardiac outflow and increase systemic vascular resistance, which results from arterial dysfunctions and atherosclerosis [17]. It seems that the connecting link between hypothyroidism and cardiometabolic risk is lipid abnormalities. In this study, it was aimed to find the association between non-HDL-C and cardiometabolic risk factors in patients with hypothyroidism.

## Materials and methods

In this case-control study, a total of 120 subjects were enrolled, comprising 60 diagnosed hypothyroidism patients and 60 age-matched healthy controls. The subjects, aged between 30 and 65 years, were selected from the Outpatient Department (OPD), Department of Medicine, University Medical Hospital. Detailed demographic, medical, and family histories were collected from each subject, with written informed consent.

The inclusion criteria for cases were individuals diagnosed with hypothyroidism, identified by a TSH level ≥5µIU/mL, while healthy subjects without hypothyroidism served as controls. Exclusion criteria included subjects with a history of chronic diseases such as liver, kidney, or pancreatic disorders, as well as those affected by infectious diseases like hepatitis, gastrointestinal tract infections, and tuberculosis. Pregnant and lactating women were also excluded from the study.

Anthropometric parameters including waist circumference (WC) and body mass index (BMI) were measured to assess physical characteristics. WC was measured using a flexible tape around the waist with minimal clothing, reported in centimeters (cm), while BMI was calculated as weight in kilograms (kg) divided by height in square meters (m²). Systolic and diastolic blood pressures (SBP and DBP) were measured using an automated blood pressure monitor based on the Oscillometry principle (Omron HEM 7120).

Sample collection involved obtaining 5 mL of venous blood from each subject under aseptic conditions after a fasting period of at least 10 hours. The blood was collected in plain vials, allowed to clot at room temperature for 15 minutes, and then centrifuged at 1000 rpm for 10 minutes to separate the serum. The serum samples were stored at 20ᵒC for further laboratory investigations.

Laboratory investigations were carried out using commercially available kits. The lipid profile and fasting blood sugar (FBS) were assessed using an Erba Chem-7 Biochemistry Semi-auto analyzer. The thyroid profile was evaluated using a Biomerieux Mini Vidas kit based on enzyme-linked fluorescence immunoassay (ELFA). Low-density lipoprotein cholesterol (LDL-C), very low-density lipoprotein cholesterol (VLDL-C), and non-high-density lipoprotein cholesterol (non-HDL-C) were calculated using Friedewald’s Formula: (LDL-C=TC-HDL-C-TG/5) and (Non-HDL-C=TC-HDL-C).

Statistical analysis was performed using IBM-SPSS software (version 20.0), Graph Pad (Prism 6.0), and Microsoft Excel (version 2013). The data were expressed as mean ± standard deviation. An unpaired t-test was conducted to compare study parameters between cases and controls, while Karl Pearson’s correlation analysis determined relationships between variables among cases. Regression analysis was carried out specifically among cases. A p-value of <0.05 was considered statistically significant.

## Results

The mean values of BMI, WC, FBS, TSH, total cholesterol (TC), triglycerides (TG), non-HDL-C, LDL-C, VLDL-C, SBP, and DBP were significantly higher in cases compared to controls (p<0.001). However, the mean values of T3, T4, and high-density lipoprotein cholesterol (HDL-C) were significantly lower in cases compared to controls (p<0.001), as depicted in (Table 1).

**Table 1:** Baseline characteristics of cases and controls Data was represented as mean ± standard deviation. p<0.05 was considered statistically significant BMI: body mass index, WC: waist circumference, FBS: fasting blood sugar, T3: triiodothyronine, T4: thyroxine, TSH: thyroid-stimulating hormone, TC: total cholesterol, TG: triglyceride, HDL-C: high-density lipoprotein cholesterol, LDL-C: low-density lipoprotein cholesterol, VLDL-C: very low-density lipoprotein cholesterol, SBP: systolic blood pressure, DBP: diastolic blood pressure

Parameters	Reference range	Case (n=60)	Control (n=60)	P-value
Age (years)	30-65 years	40.32±8.62	42.47±8.66	0.1755
BMI (kg/m^2^)	18.5-24.9 kg/m^2^	26.35±3.85	22.41±2.12	<0.0001
WC (cm)	≤90 cm	90.44±6.30	83.67±4.66	<0.0001
FBS (mg/dL)	70-100 mg/dL	101.33±21.46	91.73±14.18	0.0046
T3 (nmol/L)	1.2-2.7 nmol/L	0.91±0.22	2.05±0.87	<0.0001
T4 (nmol/L)	57-148 nmol/L	86.87±15.64	127.25±54.76	<0.0001
TSH (µIU/mL)	0.5 to 5.0 µIU/mL	12.06±1.04	2.18±0.90	<0.0001
TC (mg/dL)	≤200 mg/dL	204.11±31.34	124.54±20.53	<0.0001
TG (mg/dL)	≤150 mg/dL	171.95±53.35	123.17±35.34	<0.0001
HDL-C (mg/dL)	≥40 mg/dL	44.32±8.58	49.02±6.33	0.0009
NON-HDL-C (mg/dL)	≤130 mg/dL	152.36±38.10	75.86±24.42	<0.0001
LDL-C (mg/dL)	≤100 mg/dL	126.90±34.39	50.89±19.57	<0.0001
VLDL-C (mg/dL)	20-30 mg/dL	34.39±10.67	24.63±7.07	<0.0001
SBP (mmHg)	≤120 mmHg	141.93±13.58	122.95±5.73	<0.0001
DBP (mmHg)	≤80 mmHg	94.15±9.31	84.78±5.47	<0.0001

**Figure 1:**
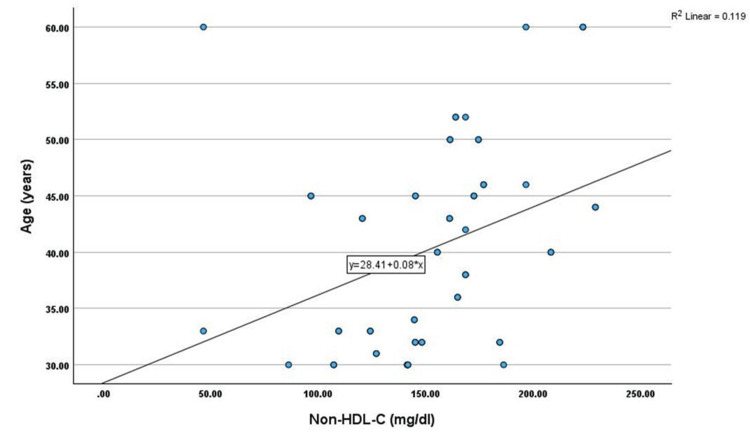
Correlation between age (years) and non-HDL-C (mg/dL) HDL-C: high-density lipoprotein cholesterol

**Figure 2:**
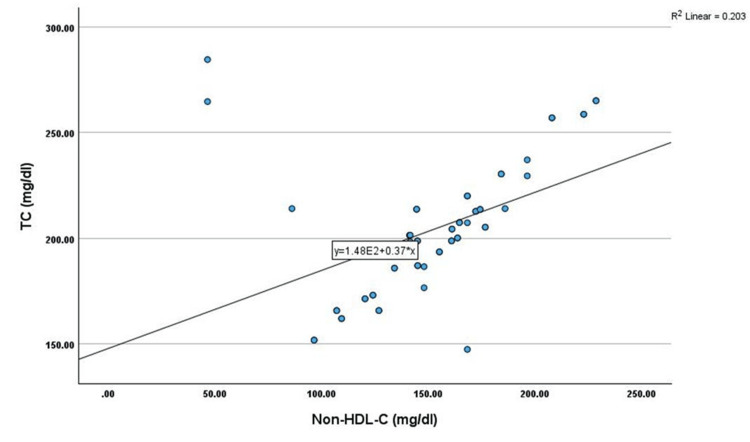
Correlation between TC (mg/dL) and non-HDL (mg/dL) TC: total cholesterol, HDL-C: high-density lipoprotein cholesterol

**Figure 3:**
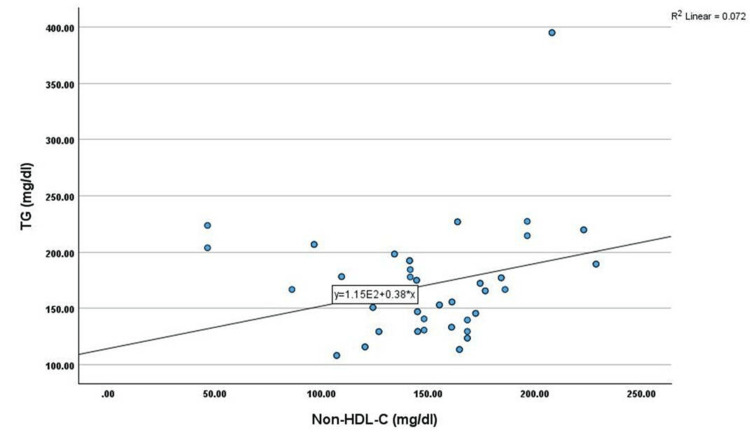
Correlation between TG (mg/dL) and non-HDL-C (mg/dL) TG: triglyceride, HDL-C: high-density lipoprotein cholesterol

**Figure 4:**
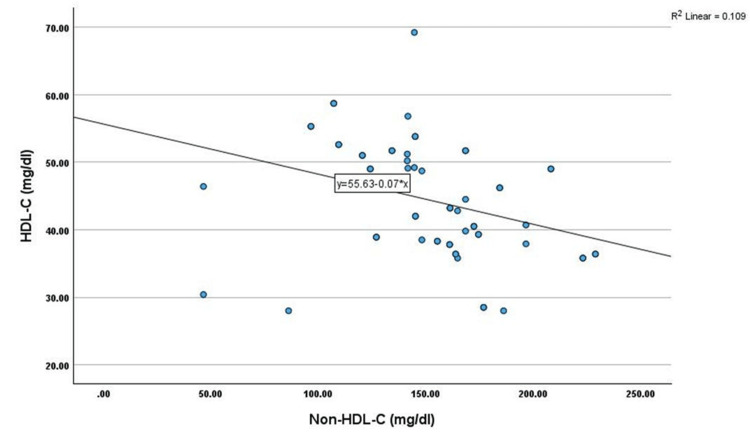
Correlation between HDL-C (mg/dL) and non-HDL-C (mg/dL) HDL-C: high-density lipoprotein cholesterol

**Figure 5:**
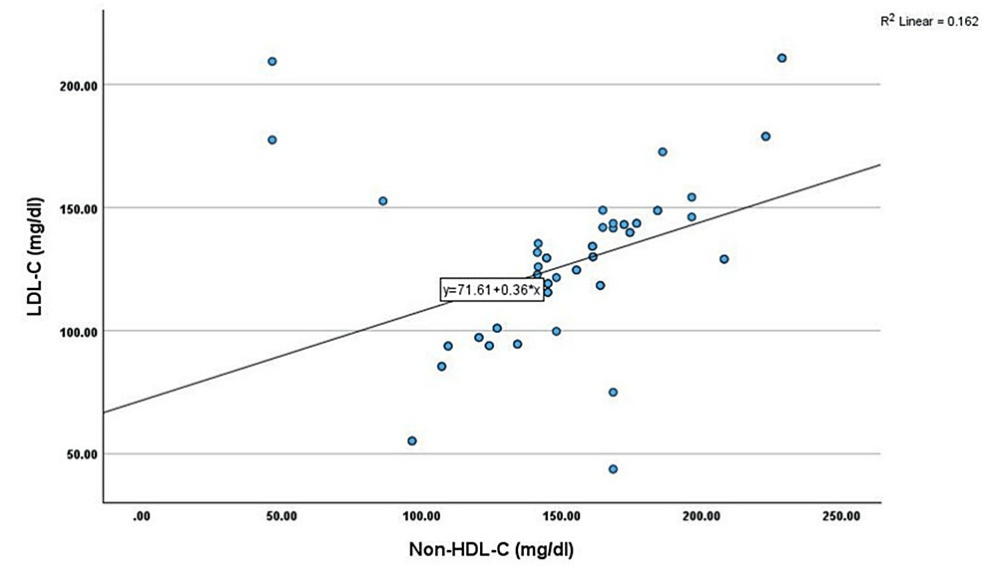
Correlation between LDL-C (mg/dL) and non-HDL-C (mg/dL) LDL-C: low-density lipoprotein cholesterol, HDL-C: high-density lipoprotein cholesterol

**Figure 6:**
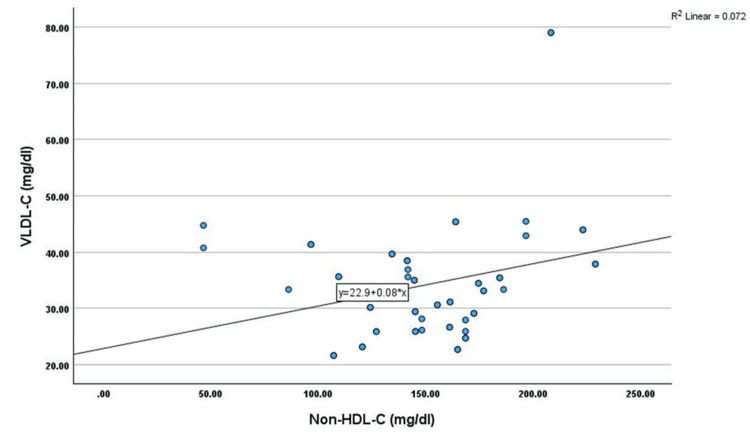
Correlation between VLDL-C (mg/dL) and non-HDL-C (mg/dL) VLDL-C: very low-density lipoprotein cholesterol, HDL-C: high-density lipoprotein cholesterol

Correlation analysis showed that non-HDL-C has shown a significant positive correlation with age (r=0.345, p<0.01), TC (r=0.451, p<0.01), TG (r=0.269, p<0.05), LDL-C (r=0.402, p<0.01), and VLDL-C (r=0.269, p<0.05) among cases. However, non-HDL-C has shown a significant negative correlation with HDL-C (r=-0.330, p<0.05) among cases, as shown in Table 2 and Figures 1-6.

**Table 2:** Correlation of non-HDL-C with different parameters among cases Data was represented as correlation coefficient (r), which is unit-free and ranges from -1 to +1. **Correlation is significant at the 0.01 level (two-tailed) *Correlation is significant at the 0.05 level (two-tailed) BMI: body mass index, WC: waist circumference, FBS: fasting blood sugar, T3: triiodothyronine, T4: thyroxine, TSH: thyroid-stimulating hormone, TC: total cholesterol, TG: triglyceride, HDL-C: high-density lipoprotein cholesterol, LDL-C: low-density lipoprotein cholesterol, VLDL-C: very low-density lipoprotein cholesterol, SBP: systolic blood pressure, DBP: diastolic blood pressure

Variables	Age	BMI	WC	SBP	DBP	FBS	TC	TG	HDL-C	LDL-C	VLDL-C	T3	T4	TSH
Non-HDL-C	0.345^**^	-0.162	0.071	-0.032	0.117	0.101	0.451^**^	0.269^*^	-0.330^*^	0.402^**^	0.269^*^	-0.104	-0.124	0.252

Regression analysis showed that non-HDL-C significantly predicted cardiometabolic risk in patients with hypothyroidism (F (13,46)=3.500, p<0.001). The R2=0.497 depicts that model 1 explains 49.7% of the variation in hypothyroidism due to non-HDL-C, as shown in Tables 3, 4.

**Table 3:** Regression analysis model summary among cases The R square of 0.497 reveals that 49.7% of the variability observed in the target variable is explained by the regression model. p<0.05 was considered statistically significant. BMI: body mass index, WC: waist circumference, FBS: fasting blood sugar, T3: triiodothyronine, T4: thyroxine, TSH: thyroid-stimulating hormone, TC: total cholesterol, TG: triglyceride, HDL-C: high-density lipoprotein cholesterol, LDL-C: low-density lipoprotein cholesterol, VLDL-C: very low-density lipoprotein cholesterol, SBP: systolic blood pressure, DBP: diastolic blood pressure

Model summary									
Model	R	R square	Adjusted R square	Std. error of the estimate	Change statistics				
					R square change	F change	df1	df2	Sig. F change
1	0.705^a^	0.497	0.355	30.85453	0.497	3.500	13	46	<0.001
^a^Predictors: (constant), TSH, HDL-C, SBP, T3, WC, T4, VLDL-C, Age, BMI, TC, DBP, FBS, LDL-C									

**Table 4:** Regression analysis coefficients among cases BMI: body mass index, WC: waist circumference, FBS: fasting blood sugar, T3: triiodothyronine, T4: thyroxine, TSH: thyroid-stimulating hormone, TC: total cholesterol, TG: triglyceride, HDL-C: high-density lipoprotein cholesterol, LDL-C: low-density lipoprotein cholesterol, VLDL-C: very low-density lipoprotein cholesterol, SBP: systolic blood pressure, DBP: diastolic blood pressure

Coefficients^a^								
Model		Unstandardized coefficients		Standardized coefficients	t	Sig.	95.0% confidence interval for B	
		B	Std. error	Beta			Lower bound	Upper bound
1	(Constant)	-41.768	120.699		-0.346	0.731	-284.722	201.186
	Age	1.746	0.725	0.395	2.408	0.020	0.287	3.206
	BMI (years)	-3.269	1.497	-0.330	-2.184	0.034	-6.281	-0.256
	WC (cm)	2.358	0.903	0.390	2.612	0.012	0.541	4.175
	SBP (mmHg)	-0.751	0.410	-0.268	-1.831	0.074	-1.577	0.075
	DBP (mmHg)	0.029	0.654	0.007	0.045	0.965	-1.288	1.346
	FBS (mg/dL)	-0.260	0.310	-0.146	-0.839	0.406	-0.883	0.363
	TC (mg/dL)	0.439	0.311	0.360	1.410	0.165	-0.188	1.066
	HDL-C	-0.494	0.748	-0.111	-0.661	0.512	-2.000	1.011
	LDL-C (mg/dL)	0.065	0.281	0.059	0.233	0.817	-0.500	0.631
	VLDL-C (mg/dL)	-0.159	0.560	-0.044	-0.283	0.778	-1.286	0.969
	T3 (nmol/mL)	-22.749	20.739	-0.131	-1.097	0.278	-64.494	18.996
	T4 (nmol/mL)	-0.168	0.301	-0.069	-0.560	0.578	-0.774	0.437
	TSH (µIU/mL)	7.589	4.529	0.207	1.676	0.101	-1.527	16.706
^a^Dependant variable: non-HDL-C								

## Discussion

The results indicate that the mean values of BMI, WC, FBS, TSH, TC, TG, non-HDL-C, LDL-C, VLDL-C, SBP, and DBP were significantly higher in cases compared to controls. However, the mean levels of T3, T4, and HDL-C were significantly lower in cases than in controls. Correlation analysis showed that non-HDL-C has a significant association with age and lipid abnormalities in patients with hypothyroidism. Regression analysis indicated that non-HDL-C significantly predicts cardiometabolic risk factors in patients with hypothyroidism.

Gutch et al. reported that levels of serum LDL-C, VLDL-C, TG, TC, and TSH were found significantly elevated in cases than in controls. However, levels of HDL-C, T3, and T4 were found significantly lower in cases than in controls. It was further suggested that thyroid functions and obesity are interlinked because thyroid hormones regulate metabolic pathways and energy expenditure [18]. A study conducted by Gonzalez et al. reported that 96.1% of hypothyroid patients have dyslipidemia and mostly have low HDL-C and high TG. It was further suggested that hypothyroidism with obesity is a significant risk factor for the development of dyslipidemia [19].

It was reviewed that lipid abnormalities and elevated TSH are strongly associated. The degree of lipid abnormality is directly proportional to the degree of elevation of TSH. It was further suggested that age, gender, and BMI may influence the pattern of lipid abnormalities. TSH is mainly elevated with an increase of LDL-C in patients with hypothyroidism. This indicated that lipid abnormalities are associated with cardiometabolic risk factors in patients with hypothyroidism [20]. In addition, depressed females with hypothyroidism are at greater risk for CVD [11].

Cappola et al. reviewed that thyroid hormone T3 induces cholesterol biosynthesis initiation enzyme HMG-CoA reductase and up-regulates LDL-C receptors that slow down the clearance of LDL-C in patients with hypothyroidism. Thyroid hormones also affect the activity of cholesterol degradation. First step enzyme 7α-hydroxylase regulates the rates of excretion of bile acid and fecal cholesterol. It was indicated that hypothyroidism is associated with high levels of LDL-C and adverse effects in the number, size, and oxidation of LDL-C [21]. Deficiency of T3 and T4 can increase TG levels by inhibiting the activity of the enzyme lipoprotein lipase [22]. A study reported that hypothyroidism patients who have serum TSH (>10 mIU/L) also have high levels of small and dense LDL-C that have more atherogenic characteristics [23]. In addition, a meta-analysis study by Rudoni et al. indicated that hypothyroidism was linked with chronic heart disease (CHD) risk factors. This study further reported that patients with hypothyroidism who have serum TSH levels (≥10 mIU/L) were associated with CHD risk factors and those who have serum TSH levels (≥7 mIU/L) were more prone to CHD-associated mortality [24]. The incidence of heart failure (biventricular) was found high in patients with untreated hypothyroidism who have serum TSH levels (≥10 mIU/L) because of abnormal hemodynamic functions. This study further suggested that thyroid hormone supplementation and restoration therapy may reverse the physiological abnormalities and maintain the hemodynamic functions of the heart [25]. A US population-based cohort study reported that high levels of serum TSH were associated with increased cardiometabolic risk and all-cause mortality [26]. It was suggested that the development of atherosclerotic CVD in patients with hypothyroidism is mediated by hyperlipidemia because hyperlipidemia is strongly influenced by changes in serum T3, T4, and TSH levels [13].

The present study showed that non-HDL-C has a significant positive correlation with age, TC, TG, LDL-C, and VLDL-C in patients with hypothyroidism. Non-HDL-C may act as a novel marker for the prognosis of insulin resistance and atherogenic CVD and coronary artery disease (CAD) [27]. Nine-year follow-up cohort study reported that elevated levels of non-HDL-C were linked with CVD than the levels of LDL-C [28]. In addition, non-HDL-C is strongly associated with increased risk factors for metabolic syndrome. Non-HDL-C may act as potential predictors for metabolic syndrome and its associated complications [29].

An epidemiological study conducted by Ghosh et al. reported that a sedentary lifestyle increases the risk factors for hypertension, diabetes, and hypothyroidism in obese individuals [30]. In addition, hypothyroid patients have shown a reduction in vascular resistance. Abnormalities in thyroid hormones and blood pressure may be the leading cause mechanisms in renal function alternations in hypothyroid patients [31]. It has been recommended that clinical management of thyroid hormones and lipid profile are important factors to reduce/reverse hypothyroidism-associated cardiometabolic risk factors [20]. 

The limitations of the study include its relatively small sample size, which may restrict the generalizability of the findings to a broader population. Further studies are required to confirm our findings and strengthen the hypothesis that hypothyroidism is strongly associated with cardiometabolic risk factors.

## Conclusions

The results indicated that the mean values of BMI, WC, FBS, TSH, TC, TG, non-HDL-C, LDL-C, VLDL-C, SBP, and DBP were significantly higher in cases compared to controls. Conversely, the mean levels of T3, T4, and HDL-C were found to be significantly lower in cases than in controls. Non-HDL-C has shown a significant association with age and lipid abnormalities in patients with hypothyroidism. Hence, non-HDL-C significantly predicts cardiometabolic risk factors in patients with hypothyroidism.
